# Migration Status, Internet Use, and Social Participation among Middle-Aged and Older Adults in China: Consequences for Depression

**DOI:** 10.3390/ijerph17166007

**Published:** 2020-08-18

**Authors:** Qian Liu, Haimin Pan, Yuanyuan Wu

**Affiliations:** 1 School of Public Administration, Hunan Normal University, Changsha 410081, China; liuqian67520@126.com (Q.L.); wyy13466847694@163.com (Y.W.); 2 Department of Sociology, Zhejiang University, Hangzhou 310058, China

**Keywords:** migration status, internet use, social participation, depressive symptoms, middle-aged and older adults

## Abstract

This study aimed to examine the underlying relationship between migration status and depressive symptoms among middle-aged and older adults in China. Data were derived from three waves of panel data (2011, 2013, and 2015) from the China Health and Retirement Longitudinal Study. Two-level regression models and generalized structural equation modeling were run to fit the data. The results showed that migration status of the respondents could ameliorate their depression (β = −0.02, *p* < 0.01), so did internet use (β = −0.02, *p* < 0.001), and social participation (β = −0.06, *p* < 0.001). The indirect effects of migration status on depression through internet use and of internet use on depression through social participation existed. The effects of migration status, internet use, and social participation in decreasing depression were discussed. Provided the associations among migration status, internet use, social participation, and depression, attention should be paid on increasing protective aspects of migration among middle-aged and older adults, such as internet use and social participation, to enhance their mental health.

## 1. Introduction

Along with the development of urbanization is a growing amount of internal migrant population in mainland China. Urbanization somewhat contributes to inequalities in physical and socio-economic conditions among different places, and some people move to the places with more resources and better public service for a prospective life. This spatial redistribution is characterized by prominent urban-orientation [1], in addition to other forms of internal migration in small proportion (e.g., city-to-city, rural-to rural). Those who live in a place but have no local Hukou would be preferably called “floating population” [2]. Hukou is a registration system that has been used since the Shang dynasty. This registration system officially records everyone’s details on personal information (e.g., name, gender, birthdate, family, marriage) and ties local public service (e.g., education, healthcare, employment), based on individuals’ basic personal information. According to the “China Floating Population Development Report 2018” issued by the National Health and Health Commission, the total floating population in 2015 was 247 million, and the elderly floating population was 13.04 million, accounting for 5.3% of the total floating population. Moreover, the number of the elderly floating population in mainland China present an increasing trend year by year, with the average annual growth rate reaching about 6.6% [3]. It is postulated that the mobility of the elderly has become normalized in China’s urbanization development, and the elderly floating population has become an important stream of China’s floating population. In particular, in mainland China, a large number of middle-age people under 60 years older take over the role of caregivers in intergenerational interaction [4]. For one thing, the unique inclination in traditional Chinese culture advocates that taking care of grandchildren is seemingly an obligation for the generation of grandparents. For another, the efforts and time offered by the parents is so limited due to the high pressure from working and living that assistance from grandparents as caregivers in providing care for grandchildren is commonly accepted and applicable [5]. As for the middle-aged and elderly people, another major reason for migration is the demand for being taken care by their adult children, aside from their main impetus to assist in taking care of grandchildren [6]. It is assumed that middle-aged and elderly people occupy a sizable proportion of migrant population owning to cultural and family preference. In spite of the underlying rationales for migration among middle-to-older adults, issues regarding their mental health are concerning.

### 1.1. Migration and Mental Health

Migration is a process of geographic movement across specified boundaries for establishing a new residence [7]. In effect, migration today is a burning social issue and a long-lasting stressful process that can increase negative health outcomes [8]. As for the migration population of middle-to-older age, migration is often accompanied by types of stresses. Which could lead to poor mental health and changes in behavior patterns [8], though many positive aspects of migration in this group are plausible as well (e.g., higher quality of life; interaction with family members) [5]. As one of the dominant theories that explain the relation between environmental experience and individuals’ mental health, the stress process theory identifies stresses to be different based on the extents of their impacts [9,10]. Besides, this theory asserts that stress exposure could induce people’s responses to these stresses and develop health outcomes as a result. During the stress-response process, personal and social resources and coping strategy are also highlighted in this theory [9,10]. By referring to the stress process model, it is sensible that migration actually can be viewed as a primary stressor, which leads to sequent secondary stressors and types of stress outcomes. After the middle-aged and elderly people enter a strange place from their initial domiciles, they are confronted with many challenges. For instance, language is a primary barrier for them to adapt to the daily life in host setting [1]. Likewise, they are often forced to build up their new social network since they break away from their original social circles and relations after migration. However, it is true that social networks, especially, neighborhood network, matter most for the elderly migrants’ mental health [11]. Their decreasing economical and physical capacity, as compared to younger migrants, for overcoming the migration-related hardship could additionally jeopardize their adaption to the host society and finally mental health [12]. From the macro perspective, institutional barriers, such as those in health insurance, housing, and employment, could also prevent them from getting attached to the new habitat [13]. With those stresses faced, this group of migrant people in middle and older age are vulnerable to many mental disorders, for example, depression [12], and would have higher level of depression and more difficulties in activities of daily living than non-immigrant elders [14]. To be noted, the stress process model also highlights coping resources, either personal factors (e.g., self-esteem) or social coping factors (e.g., social participation), that could be taken advantage of to deal with experienced stressors [15]. In this line, this study centered on two factors, namely, internet use, and social participation, based on their breadth and applicability in the field of mental health research.

### 1.2. Migration, Internet Use, and Mental Health

According to the 44th China Statistical Report on Internet Development in 2019, there have been 854 million netizens in China, with 99.1% of them using mobile phones to surf the internet [16]. In current days, internet use becomes indispensable in most people’s daily lives, for example, buying daily necessities (e.g., Alipay), and communicating with family members, friends, and others (e.g., WeChat). Migrants may also use telecommunications via the internet to meet such daily needs for life. Furthermore, migrant workers would use the internet as a strategy in response to alienation, discrimination, and restrictions on action they face in adaption process [17]. Older migrants could use the internet to reconcile their feeling of loneliness by engaging themselves in activities and interaction with others and strengthen their connectedness with social members, for example, by communicating with their long-distance older friends and make new friends nearby, whereby they could increase independence and handle challenges caused by not only aging issues but migration [18]. For instance, entertainment-oriented use of the internet could provide migrants of all ages with emotional support and a strong sense of their local identity, and online games allow them to relieve the pressure of life [19]. Overall, internet use, as for migrant population, including those in middle aged and older adults, could prompt them to adjust hinders in their psychological and social health. The function of internet use has been growing diverse, sharply, and internet use has become the integral part of the lives of migrants in different age cohorts. Despite the large-scale application of internet use among the general migrant population, its width of application in middle-aged and elderly people is pending, so is its influence in their mental health. Actually, the question on what influence internet use could make in middle-aged and older adults’ mental health is of value in that services related could be promoted accordingly.

In effect, the positive effect of internet use on mental health has received attention elsewhere. Previous studies showed that higher levels of internet use were significantly associated with better life satisfaction, reduced loneliness and depressive symptoms, and psychological well-being among older adults [20,21,22]. This benefit of internet use could be attributed by the increased social support, a sense of empowerment, feeling of independence, and incremental physical health among older migrants [20,23]. Likewise, a qualitative study illustrated that participation in social networking website helped foster a supportive environment and a sense of increased connectivity with surrounding people [24]. Therefore, the importance of technology use has been elevating in high-speed based on its intense and broad coverage in people’s life, including middle-aged and elderly people in migration. Among the factors that help to bring about the link of internet use to mental health, activity participation is a prominent one that is prevalent in mental health research, but is sparsely applied in migrant people in association with internet use. 

### 1.3. Internet Use, Social Participation, Mental Health

More active internet use in old age, in effect, has a strong positive correlation with the number of various leisure activities [25]. It is said that existing computer technology and the internet act as important locations of contemporary leisure activity [26]. This new space for leisure participation renders an updated pattern of leisure activities experiences. In addition, voluntary work and participation, for example, often requires internet use for information delivery and activity design and control [27]. Thus, internet technology serves as a media for people to experience activities. Moreover, internet use could be viewed as a tool of facilitating social activities for people to participation. Internet is treated as a resource that enhances the community participation and trust among community-dwelling residents by which many resource-based frameworks could be built up [28,29]. As for older adults, this potential of internet use could be particularly embodied in easier accesses to reach people and meet new people, as well as in increasing the quality of communication with others and, thereby, decreasing their loneliness [30]. Accordingly, the forms of activities have become pluralistic through internet, and the online activities broaden the range of choices for people to participate in addition to the offline activities. In this sense, it is postulated that internet use is profitable for activity participation among Chinese migrant population in middle-to-older age. However, to the best knowledge, investigation in this line is unavailable.

The concept of social participation is under a going debate on its definition, with terms, like, participation, social engagement, social involvement, social participation, and social needs being used interchangeably [31]. Generally, social participation refers to “performance of people in actual activities in social life domains through interaction with others in the context in which they live” [32]. Evidence has demonstrated that social participation in activities is beneficial for mental health in both Western and China populations, including migrant people [33,34,35]. This association could stem from reduced risk of social isolation and enhanced emotional intimacy, better cognitive function and a sense of being valued among people in later life [36,37]. It is presumed that those notions can also be applicable to migrant people in middle-to-older adults in that such benefits would be come into surface when they take part in social activities. Although the effects of social participation sometime is conditioned, for example, on the personal traits (e.g., age, gender) and the types of activities [38,39], the exploratory observation on the role of social participation in promoting mental health among middle-to-older migrant people in China is primary and worthwhile.

### 1.4. This Current Study 

**Figure 1:**
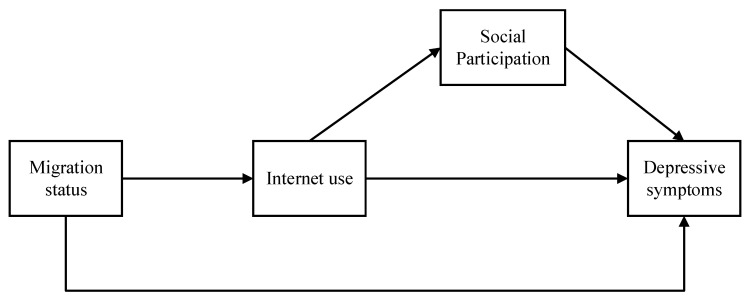
The proposed model including stressors, coping resources, and stress outcome.

According to the stress process model, understanding the potential stress-coping patterns of resources is important [40]. Based on the literature reviewed and mentioned as above and the stress process model, a proposed model was developed as depicted in Figure 1. In this model, migration was viewed as a primary stressor, which could finally lead to many diverse health outcomes (stress outcomes). Depression was examined as an outcome of migration in this study. Meanwhile, internet use and social participation were the two coping factors that would diminish the negative effect of migration, and particularly, internet use served as a mediator in the relationship between migration status and depressive symptoms and social participation in the relationship between internet use and depressive symptoms. Accordingly, many hypotheses are proposed:

**Hypothesis** **1** **(H1).** *Migration would increase depression among middle-aged and older people in China.*

**Hypothesis** **2** **(H2).** *Migration would increase the frequency of internet use among middle-aged and older people in China.*

**Hypothesis** **3** **(H3).** *Internet use would mediate the relationship between migration and depression, and increase the degree of social participation among middle-aged and older people in China.*

**Hypothesis** **4** **(H4).** *Social participation would mediate the relationship between internet use and depression among middle-aged and older people in China.*

## 2. Methods

### 2.1. Sample

The sample of this study were drawn from “The China Health and Retirement Longitudinal Study (CHARLS)”, with three waves of data included (2011, 2013, and 2015). The CHARLS survey was a nationally representative longitudinal study of Chinese people over 45-year old and their spouses, and covered wide domains, such as demographics characteristics, family structure, income, health care and insurance, health status and functioning, and expenditure and assets [41]. The baseline data consisted of 17,708 respondents in 10,257 households from 450 villages/urban communities in 150 counties/districts across 28 provinces in China during the period of 2011–2012 by using the multi-stage stratified probability-proportional-to-size (PPS) sampling method. The response rate of completing at least one point of data collection was 80.5% [42]. Data collection was conducted every 2 years and the number of original respondents who participated in 2013 and 2015 waves were 15,104 and 14,126, respectively. In 2013, a new sample of 3508 respondents was replenished. This part of sample had 1701 respondents left in 2015. In 2015, a total of 3826 respondents was replenished. The major reason for attrition is mortality. The CHARLS survey obtained ethical approval from the Biomedical Ethics Review Committee of Peking University (IRB00001052-11015) [43]. Observations with missing values in the analytic variables were deleted, and the final longitudinal sample includes 40,789 observations, which were nested within 18,511 respondents.

### 2.2. Measures

#### 2.2.1. Dependent Variables

Depressive symptoms. Depressive symptoms were measured by the ten-item Center for Epidemiologic Studies-Depression scale (CES-D) [44]. Five of the depression items signified somatic symptoms (being bothered by things, having trouble to concentrate, needing an effort to do everything, having poor sleep, feeling unable to go on); three items indicated feelings of negative affect (feeling depressed, feeling fearful, feeling lonely) and the remaining two items revealed feelings of positive affect (feeling hopeful, feeling happy). Respondents were asked to report the intensity they felt regarding each symptom in the past week (0 = rarely or none of the time; 1 = not too much time; 2 = sometimes or half the time; 3 = most of the time). Furthermore, we reversed the coding of two positive affect items, and then summed up the scores of the ten items. The depression scores ranged from 0 to 30, with a higher score indicating severer depressive symptoms. The reliability coefficients for this scale ranged from 0.75 to 0.80 across three waves.

#### 2.2.2. Independent Variables

##### Migration Status

Middle-aged and older migrants referred to individuals aged 45 years and over who had no local residency status. Migration status was ascertained by asking respondents about their current Hukou status and residential location. The migration status was assessed by a single item: “Where is your current Hukou?” It was dummy coded (1 = migration, 0 = non-migration). Migrants were defined as those who lived in the different village with Hukou location; non-migrants were defined as those who lived in the same village with Hukou location. 

##### Internet Use

Internet use was assessed by a single item: “Have you used the Internet in the last month?” The options for respondents to select were 0 = no, 1 = yes.

##### Social Participation

Social participation was measured with six items. Respondents reported whether they took part in the following activities in the last month (1 = yes, 0 = no): (a) interacted with friends, (b) played Mahjong, played chess, played cards, or went to community club, (c) provided help to family, friends, or neighbors who do not live with you, (d) went to a sport, social, or other kind of club, (e) took part in a community-related organization, (f) did voluntary or charity work. Afterwards, we added up the scores of six items together, with higher scores indicating higher levels of social participation. 

#### 2.2.3. Control Variables

Control variables included age, gender (male, female), marital status (married, unmarried), education (in years), retired or not (yes, no), household per capita consumption expenditure (RMB), living with children or not (yes, no), Hukou (agricultural, non-agricultural), and the wave involved. Categorical variables were dummy coded, with each of them having the following reference categories: female for gender, unmarried for marital status, and non-agricultural for Hukou. Household per capita consumption expenditure in the previous year was computed in the form of the common logarithm of household per capita consumption expenditure in the previous year plus 1(Yuan), e.g., log (household per capita consumption expenditure in the previous year +1). Furthermore, we constructed a variable of time interval manifested as 1 (2011) to 3 (2015) due to intervals between each data collection were of similar length.

### 2.3. Data Analysis Strategy

We used a two-level model with maximum likelihood estimation-using xtreg commend in Stata Statistical software Version 15 (Stata 15.) for analyzing longitudinal data with attrition. All of the variables were included in model at either level 1 (time varying) or level 2 (time invariant). The two-level regression models were specified as random intercept models, allowing the specific intercepts to vary from individual to individual. Thus, individual characteristics were included in the models, and accordingly there were additional unmeasured effects at level 2 that explained intrapersonal variance. Model 1 included migration status and control variables. In model 2 and Model 3, we added sequentially two variables (internet use and social participation) to Model 1 in order of entry. The ordering is a good way to see how the added variables explained the previous main variables. Of interest was the degree to which internet use and social participation mediated the effects of respondents’ migration status on their depressive symptoms.

Generalized structural equation modeling (GSEM) was used to examine the pathways of the effect of older adults’ migration status on their level of depressive symptoms. This study chose GSEM for data analysis, since it allows for continuous, binary, count, and multinomial modeling, and provides estimates of robust standard errors [45,46]. Through this study could we confirm a speculated pathway where respondents’ migration status affected internet use, social participation, and ultimately, depressive symptoms.

This study used the “gsem” commend in Stata 15 when applying generalized structural equation models to simultaneously examine the direct and indirect effects of migration status, internet use, and social participation on the level of depressive symptoms. All of these variables in our models were observable variables. Owing to the fact that GSEM procedure did not produce goodness-of-fit indices, we only reported Akaike Information Criterion (AIC) and Bayesian Information Criterion (BIC) values of the corresponding models [45,47]. Although the models in this study could not ascertain the causal association, it could examine if the assumptive causal pathways are plausible or not. Therefore, these results should be explained as correlations.

## 3. Results 

As shown in Table 1, the average score of depressive symptoms was 8.10 (SD = 6.20); 11.84% of respondents were migrants, and 4.27% of them used the internet. The mean of social participation in middle-to-older aged adults was 0.82 (SD = 0.96). The other analytic variables’ information were presented in Table 1.

Table 2 showed the three regression models predicting depressive symptoms. Model 1 revealed that middle-aged and older adults who were migrants less likely to have depressive symptoms than those who were not. Middle-aged and older adults who were younger, male, married, higher education, non-retired, and having non-agricultural Hukou tended to be less depressive than their counterparts. As time went on, middle-aged and older adults’ depressive symptoms became weakened. In Model 2, with internet use added, the result showed that middle-aged and older adults who used the internet were less likely to have depressive symptoms than their counterparts. Model 3. including social participation variable, additionally revealed that social participation was significantly negatively associated with depressive symptoms. 

**Figure 2:**
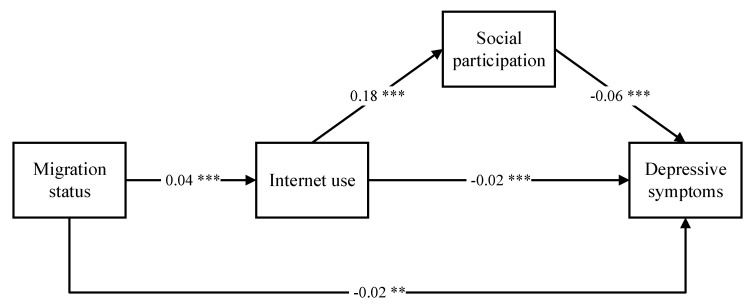
Generalized structural equation model of the effect of migration status, internet use, and social participation to depressive symptoms. Note: * *p* < 0.05, ** *p* < 0.01, *** *p* < 0.001.The model control for age, gender, marital status, education years, retired or not, household per capita consumption expenditure in the previous year (RMB), living with children or not, Hukou, and wave, Log Likelihood (LL) = −161,245.9, Degree of Freedom (DF) = 23, Akaike Information Criterion (AIC) = 322,537.8, Bayesian Information Criterion (BIC) = 322,736.

Figure 2 showed that migration status was significantly positively related to their internet use (β = 0.04, *p* < 0.001) and negatively associated with depressive symptoms (β = −0.02, *p* < 0.01). Internet use was positively related to their social participation (β = 0.18, *p* < 0.001) and negatively related to depressive symptoms (β = −0.02, *p* < 0.001). Social participation was negatively associated with depressive symptoms (β = −0.06, *p* < 0.001). It can be seen that the effect of migration status was mediated by internet use and social participation. 

## 4. Discussion 

This study developed three two-level regression models and a pathway model to explore the underlying mechanism combining migration status, internet use, social participation, and depressive symptoms among middle-aged and older migrants in China. The results found that migration was significantly negatively related to depression, and internet use acted as a mediator in the relationship between migration status and depression. Besides, social participation served as a mediator in the association between internet use and depression. These results contributed to the theoretical and practical implications in terms of how to decrease the depression of middle-aged and older adults by increasing protective aspects of migration, internet use and activity participation.

First, our study found, to be surprised, that migration could be negatively associated with depression in middle-aged and older adults. H1 was not supported. This finding from the current study is inconsistent with the stress process model [9,10] and some previous studies [12,14]. This inconsistence may lie in the fact that the purpose for migration of middle-aged and older adults in China is different from that of Western people. It is suggested that the majority of middle-to-older migrants choose to move to the place where their adult children live for family reunion. In detail, the traditional culture of filial piety in China speaks highly of intergenerational co-residence [48]. As Confucius wrote, “While his parents are alive, the son may not go abroad to a distance” [49]. Contemporarily, family ties still feature dominantly in intricate interconnectedness in people’s life. Many previous empirical studies found that living with children may directly improve older Chinese parents’ well-being [48,50]. Furthermore, another two major impetuses of middle-aged and older adults for migration might be the assistance in taking care of grandchildren and the demand for being taken care by their adult children [6]. It means that migration might bring a chance to reunite with their families and it weights a lot in developing middle-aged and older migrants’ mental health, though other facets of stress exposure exits. Accordingly, prior studies found that family separation was the strongest risk factor of depression and accounted for a sizeable part of the heightened depressive symptoms among migrants [51,52]. Ultimately, it is assumed that the positive aspects of migration (e.g., family reunion) in influencing mental health outnumbered the negative ones of migration (e.g., broken neighborhood network) among middle-aged and older adults in China. This finding also highlighted the family to be the most important predictor of depression possibly among middle-aged and older migrants in China.

Second, our study indicated that internet use was negatively related to depression and partially mediated the relationship between migration status and depressive symptoms. These findings supported H2 and H3. It turned out that middle-aged and older migrants were more likely to use the internet than their counterparts. This finding is consistent with previous studies that the rate of internet use in migrant population surpassed that in the local population [53]. The possible reason for this phenomenon was that the internet could establish social networks without the need for local involvement [54]. Thus, in order to communicate with kin and friends in hometowns, middle-aged and older migrants are more likely to use the internet than their counterparts. We also found that internet use could ameliorate depression. This accords with the previous studies where many positive aspects of internet use were asserted among middle-aged and older migrants [20,23], such as increased social support, a sense of empowerment, feeling of independence, and incremental physical health, all of which can improve their mental health. 

Moreover, our study revealed that social participation was significantly negatively associated with depressive symptoms and served as a mediator in the relationship between internet use and depressive symptoms. This finding supported H4. The positive association between internet use and social participation was also confirmed in prior studies that internet use could improve activities participation, such as community involvement and participation in voluntary organizations [25,55,56]. More specific, middle-aged and older adults may use the internet as a media to get information of activity or a tool to design and control some activities (e.g., voluntary participation, community activity, and so on) [27], which could further facilitate their active participation in social activities. What is more, this study unveiled that social participation negatively related to depressive symptoms. This finding resonates with the previous evidence that older adults who had higher frequency of participating activities tended to have fewer depressive symptoms than their counterparts [39,57]. This phenomenon possibly results from the fact that social participation could be beneficial to obtain emotional support, increase cognitive function, sustain a positive self-concept, and improve their self-esteem, thereby contributing to good mental health in later life [36,58,59]. In sum, internet use is associated with a higher level of social participation, in turn leading to fewer depressive symptoms. 

Some limitations of this current study should be noted. First, due to the original data limitations, only migration status was included in this study. Other important lens, such as the frequency, distance, or time of migration, should be taken into consideration in future studies. Second, this study only paid attention to the effect of the number of social activities on mental health. Empirical studies showed that the effects of various types of social activities on depression were not unified [39,57,60]. Future studies could be dedicated to tease out the diversity of the effects of different types of social activities on depression. Third, although the nationally representative data were used in this study, many samples were excluded due to missing values in the key variables. Therefore, the shrinking representativeness of the results of this study warrant carefulness in interpreting the results. Fourth, measurement of internet use with a single item in this study could be oversimplified. Further research is necessary to diversify more contents of the construct, such as type of internet use, length of internet use, and so forth. Last but not least, the ameliorating effect of migration status, internet use, and social participation was not scrutinized by qualitative feedback from the respondents. With insights into the potential of these factors, understanding on the negative associations of migration status, internet use, and social participation with depression could be deepened. Many rationales for such associations could be teased out. Therefore, future studies may consider collecting some qualitative data to address these questions raised.

Despite these limitations, this study is the first attempt to test the mechanism including migration status, internet use, social participation, and ultimately, depression in later-life. It could deepen our understanding on migration by testing the underlying relationship between migration status and depression. This study also broadens the application of stress process theory and supplements its evidence in Chinese context, especially among middle-aged and older adults in China. Furthermore, this study has some practical implications. First, this research found that migration could be a protective factor for middle-aged and older adults’ mental health in China. Thus, policymakers should make and implement more appropriate policies for family migration. Second, as internet use could promote mental health among middle-to-older migrants, it will be of value and full of practical implication if policymakers help to improve the middle aged and older migrants’ ability to use the internet or develop online programs. Third, based on the positive association of social participation with depression, it is implied that social and public programs to promote social participation among middle-aged and older adults can be an effective policy to improve mental health in China. 

## 5. Conclusions

The current study emphasizes the underlying mechanisms in relationship between migration status and depression among middle-aged and older adults. The results reveal that family-oriented migration, internet use, and social participation could relieve depression among middle-aged and older adults in migration. Shaping the understanding of the mechanism between migration status and depression of Chinese middle-aged and older adults is helpful in developing tailored prevention and intervention strategies to improve the mental health of middle-aged and older migrants.

## Figures and Tables

**Table 1:** Description of Analytic Variables (*n*_old adults_ = 18,511, *n*_observations_ = 40,789).

Variable	Mean (Range) *n* (%)	SD
Dependent variable		
Depressive symptoms	8.10 (0–30)	6.20
Independent variables		
Migration status		
Migration	4829 (11.84%)	
Non-migration	35,960 (88.16%)	
Internet use		
Yes	1741 (4.27%)	
No	39,048 (95.73%)	
Social participation	0.82 (0–6)	0.96
Control variables		
Age	59.98 (45–96)	9.31
Gender		
Male	19,933 (48.87%)	
Female	20,856 (51.13%)	
Marital status		
Married	35,837 (87.86%)	
Unmarried	4952 (12.14%)	
Education years	4.95 (0–22)	4.71
Retired or not		
Yes	27,769 (68.08%)	
No	13,020 (31.92%)	
Household per capita consumption expenditure in the previous year (RMB)	8.69 (0, 12.81)	1.19
Living with children or not		
Yes	22,189 (54.40%)	
No	18,600 (45.60%)	
Hukou		
Agricultural	31,366 (76.90%)	
Non-agricultural	9423 (23.10%)	
Wave		
Wave 1	13,888 (34.05%)	
Wave 2	13,609 (33.36%)	
Wave 3	13,292 (32.59%)	

Note: SD = standard deviation.

**Table 2:** Random-effects models predicting depressive symptoms (*n*_old adults_ = 18,511, *n*_observations_ = 40,789).

Variable	Model 1	Model 2	Model 3
	β (SE)	β (SE)	β (SE)
Independent variable			
Migration	−0.31 ** (0.09)	−0.31 **(0.09)	−0.31 ** (0.09)
	CI (−0.50, −0.13)	CI (−0.49, −0.12)	CI (−0.49, −0.12)
Used the internet		−0.78 *** (0.16)	−0.59 *** (0.16)
		CI (−1.09, −0.48)	CI (−0.89, −0.28)
Social participation			−0.37 *** (0.03)
			CI (−0.43, −0.31)
Control variable			
Age	0.01 ** (0.005)	0.01 * (0.005)	0.01 * (0.004)
	CI (0.004, 0.02)	CI (0.002, 0.02)	CI (0.0002, 0.02)
Male	−1.34 *** (0.08)	−1.34 *** (0.08)	−1.35 *** (0.08)
	CI (−1.50, −1.19)	CI (−1.50, −1.19)	CI (−1.51, −1.20)
Married	−1.55 *** (0.11)	−1.56 *** (0.11)	−1.58 *** (0.11)
	CI (−1.77, −1.33)	CI (−1.78, −1.35)	CI (−1.79, −1.36)
Education years	−0.19 *** (0.01)	−0.18 *** (0.01)	−0.17 *** (0.01)
	CI (−0.20, −0.17)	CI (−0.20, −0.16)	CI (−0.19, −0.15)
Retired	−0.51 *** (0.07)	−0.51 *** (0.07)	−0.05 *** (0.07)
	CI (−0.65, −0.37)	CI (−0.65, −0.37)	CI (−0.64, −0.36)
Household per capita consumption expenditure in the previous year (RMB)	0.0002 (0.02)	0.01 (0.02)	0.02 (0.02)
	CI (−0.05, 0.05)	CI (−0.04, 0.05)	CI (−0.03, 0.07)
Living with children	−0.01(0.06)	−0.01(0.06)	−0.03(0.06)
	CI (−0.13, 0.10)	CI (−0.13, 0.10)	CI (−0.14, 0.09)
Agricultural	1.40 *** (0.10)	1.34 *** (0.10)	1.27 *** (0.10)
	CI (1.21, 1.60)	CI (1.14, 1.53)	CI (1.07, 1.46)
Wave 2	−0.45 *** (0.05)	−0.44 *** (0.05)	−0.37 *** (0.06)
	CI (−0.56, −0.34)	CI (−0.54, −0.33)	CI (−0.48, −0.26)
Wave 3	−0.22 *** (0.06)	−0.20 ** (0.06)	−0.15 ** (0.06)
	CI (−0.33, −0.11)	CI (−0.31, −0.09)	CI (−0.26, −0.04)
Constant	9.82 *** (0.43)	9.91 *** (0.43)	10.21 *** (0.43)
	CI (8.98, 10.67)	CI (9.07, 10.76)	CI (9.37, 11.06)
D.F.	11	12	13
LRχ2	2153.56 ***	2179.10 ***	2328.11 ***

Note: * *p* < 0.05, ** *p* < 0.01, *** *p* < 0.001. SE = Standard Error.
